# Putting women at the center: a review of Indian policy to address person-centered care in maternal and newborn health, family planning and abortion

**DOI:** 10.1186/s12889-017-4575-2

**Published:** 2017-07-14

**Authors:** Aradhana Srivastava, Devaki Singh, Dominic Montagu, Sanghita Bhattacharyya

**Affiliations:** 1 Public Health Foundation of India, Plot no. 47, Sector 44 Institutional Area, Gurgaon, Haryana 122002 India; 2 University of California, San Francisco, USA

**Keywords:** Maternal and newborn health, Family planning, Abortion, Policy, Person-centered care, Quality of care, India

## Abstract

**Background:**

Person-centered care is a critical component of quality care, essential to enable treatment adherence, and maximize health outcomes. Improving the quality of health services is a key strategy to achieve the new global target of zero preventable maternal deaths by 2030. Recognizing this, the Government of India has in the last decade initiated a number of strategies to address quality of care in health and family welfare services.

**Methods:**

We conducted a policy review of quality improvement strategies in India from 2005 to 15, covering three critical areas– maternal and newborn health, family planning, and abortion (MNHFP + A). Based on Walt and Gilson’s policy triangle framework, we analyzed the extent to which policies incorporated person-centered care, while identifying unaddressed issues. Data was sourced from Government of India websites, scientific and grey literature databases.

**Results:**

Twenty-two national policy documents, comprising two policy statements and 20 implementation guidelines of specific schemes were included in the review. Quality improvement strategies span infrastructure, commodities, human resources, competencies, and accountability that are driving quality assurance in MNHFP + A services. However, several implementation challenges have affected compliance with person-centered care, thereby affecting utilization and outcomes.

**Conclusion:**

Focus on person-centered care in Indian MNHFP + A policy has increased in recent years. Nevertheless, some aspects must still be strengthened, such as positive interpersonal behavior, information sharing and promptness of care. Implementation can be improved through better provider training, patient feedback and monitoring mechanisms. Moreover, unless persisting structural challenges are addressed implementation of person-centered care in facilities will not be effective.

**Electronic supplementary material:**

The online version of this article (doi:10.1186/s12889-017-4575-2) contains supplementary material, which is available to authorized users.

## Background

Patient, client or person-centered care is a critical component of quality of care for enabling adherence to treatment and maximizing health outcomes. Appropriate care, which is also a satisfying experience for the woman, is the key to sustained utilization of maternal health services by women. The Institute of Medicine (IOM) defines patient-centered care as: "Providing care that is respectful of and responsive to individual patient preferences, needs, and values, and ensuring that patient values guide all clinical decisions." [1]. Acknowledging and respecting the diversity in people’s culture, values and preferences, patient-centered care views patients or clients as unique individuals who need to be engaged as active participants in the decision making and process of care [2]. Research evidence points out that patient-centered care leads to improved health access, utilization and outcomes, especially in socio-economically, ethnically and culturally diverse regions [3]. It therefore constitutes a critical policy focus in developing country contexts where socio-economic disparities and cultural norms often affect the demand and experience of care.

Improving the quality of health services is one of the key strategies to achieve the new global target of zero preventable maternal deaths by 2030 [4]. In the last decade India has seen significant improvement in health sector development through the National Rural Health Mission (NRHM), a seven-year national health sector reform program that was launched in 2005. Efforts to expand access to emergency obstetric and newborn care, family planning and safe abortion care in recent years have led to increased utilization of facility based maternal, neonatal, family planning and abortion care services. However, the sustainability of such efforts is possible only through provision of quality services leading to demonstrable positive outcomes. The national policy explicitly states that “All government and publicly financed private health care facilities would be expected to achieve and maintain Quality Standards.” [5].

In this paper we focus on three critical areas of care – maternal and newborn health, family planning and abortion (MNHFP + A). Over the recent years, a number of national policies and strategies have been brought out to address quality of care in health and family welfare services in health facilities located in urban areas. We aim to analyze these policies and strategies in the last decade with respect to MNHFP + A, assess the extent to which they incorporate person-centered care and identify any unaddressed areas. We also suggest what measures could be taken to make policies more sensitive to person-centered care. For our analysis we have used the IOM definition of patient-centered care, discussed above.

## Methods

We conducted this retrospective analysis of policies addressing quality of care in health facilities located in India from 2005 to 2015. We used the case study approach, which explores in-depth a complex phenomenon in its real world context, enabling a full contextual definition and more policy relevant analysis [6].

### Analytical framework

**Fig. 1:**
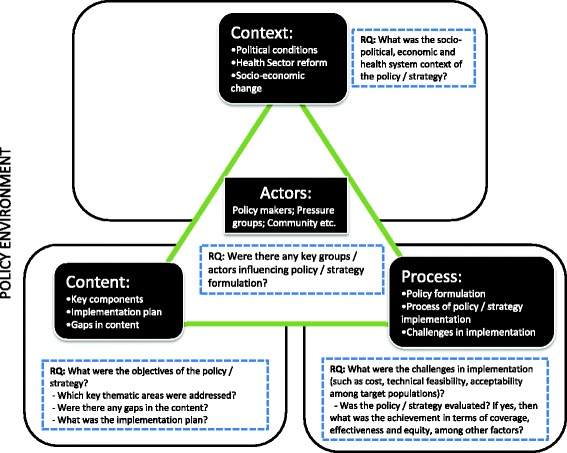
Policy Triangle Framework. [adapted from Walt & Gilson 1994]

Our analysis is based on Walt and Gilson’s policy triangle framework [7]. The policy triangle framework helped identify the contextual factors related to the policies, the people who influenced policy formulation, the policy contents and the processes whereby the policy was formulated, implemented and evaluated [Fig. 1].

Deriving from the policy triangle framework and the multiple streams theory, we asked the following research questions:

1. What was the socio-political, economic and health system context of the policy / strategy?
2. Were there any key groups / actors influencing policy / strategy formulation?
3. What was the process of policy formulation?
4. What were the objectives of the policy / strategy? Which key thematic areas were addressed? Were there any gaps in the content?
5. What was the implementation plan?
6. What were the challenges in implementation (such as cost, technical feasibility, acceptability among target populations)?
7. Was the policy / strategy evaluated? If yes, then what was the achievement in terms of coverage, effectiveness and equity, among other factors?

All research questions were analyzed through desk review of policy documents and relevant evaluations and reviews. Our analysis responded to these questions, which have been summarized in Table 1. In the paper we meld these responses into an integrated narrative of the context, policy and its associated implementation patterns, achievements and challenges.

**Table 1:** Responses to research questions based on our findings

Research Questions	Responses
What was the socio-political, economic and health system context of the policy / strategy?	High population and high poverty ratio; disparity in development indicators; absence of well-defined hierarchy of public health centers in urban areas; inequity in access of health services; quality of care is a key concern in public health service delivery.
Were there any key groups / actors influencing policy / strategy formulation?	Civil society groups born out of the movement for reproductive rights in the 1990s influenced policy making significantly and added focus on quality, patient rights and dignity.
What was the process of policy formulation?	Text drafted by the Ministry of Health and Family Welfare after multiple rounds of consultation with governmental and non-governmental stakeholders; submitted to a Committee of Parliamentarians for scrutiny, placed on website for public feedback, and final draft approved for financial support by the Cabinet Committee on Economic Affairs.
What were the objectives of the policy / strategy? Which key thematic areas were addressed? Were there any gaps in the content?	Objectives – to bring about fundamental changes in the healthcare delivery system with greater investment, decentralization and community participation. Thematic areas included improved planning, availability, access and quality of health services. Our analysis focused on the quality improvement theme. No gaps in content emerged in the analysis.
What was the implementation plan?	Public health standards were laid out and additional funding provided to help facilities achieve them. Quality certification body set up. District level system established for quality monitoring; non-governmental organizations and private sector engaged in the effort. Guidelines issued for ensuring quality of MNHFP + A care at all levels.
What were the challenges in implementation (such as cost, technical feasibility, acceptability among target populations)?	Persisting challenges included slow pace of structural improvements, availability of human resources and supplies, and lack of adherence to protocols.
Was the policy / strategy evaluated? If yes, then what was the achievement in terms of coverage, effectiveness and equity, among other factors?	No policy /strategy evaluations were available, though individual scheme evaluations and annual program reviews were available. Evidence showed that while financial incentives improved utilization, structural and human resource capacity could not be increased at the same pace, thereby affecting patient-centered care.

We used the IOM’s quality of care framework [1] to extract the quality themes covered by the various policy documents in our analysis. Quality of care comprises of three dimensions - structure, process and outcome, and quality improvement encompasses six aims – to make healthcare safe, effective, patient-centered, timely, efficient and equitable [1].

### Data collection plan

For this study, we used the definition of health policy as ‘the decisions, plans, and actions undertaken to achieve specific health care goals within a society’ [8]. The study data therefore comprised policy, strategies, programs and schemes on quality of care in MNHFP + A. Relevant documents included legislations, policy statements, strategies, program guidelines, notifications, circulars and minutes of meetings. To collect data we conducted searches of Indian government and academic websites, scientific and grey literature databases.

### Search strategy

Websites including that of the Ministry of Health, and Women and Child Development, national academic institutions, development agencies and non-governmental organizations implementing large health programs were searched. The key terms used included ‘maternal health’, ‘neonatal health’, ‘health services’, ‘mothers’, ‘newborns’, ‘family planning’, ‘abortion’, ‘quality of care’, ‘access’, ‘policy’, ‘plan’, ‘program’, ‘strategy’, ‘guidelines’ and ‘India’ in different combinations in the databases of Pubmed, Indmed (for Indian scientific journals), Popline and Google Scholar.

### Inclusion criteria

Policy documents were included if they addressed quality of care in India in at least one of the fields of maternal, neonatal health or family planning belonging to the year 2005 or later, up to February 2016. Documents in both English and Hindi were considered for inclusion, but the final documents included were in English.

### Exclusion criteria

Studies were excluded if they did not address the quality of care in facilities, were not related to MNHFP + A or were dated prior to 2005.

### Analysis plan

#### Data extraction

In the first stage of extraction the documents were summarized chronologically to describe the sequence of key milestones in the MNHFP + A quality of care policy development process. Documents were extracted electronically onto structured formats based on the policy models. The extraction was conducted by one researcher. A senior researcher then independently reviewed a sample of the documents to validate the extractions.

#### Data analysis

Thematic analysis of the data was conducted – initially the quality improvement policies were categorized into themes based on the policy triangle framework: content (maternal, newborn health, family planning or abortion care), actors, process and context. Additional themes were then identified and added, further qualifying the data into themes of care addressed by the documents (such as infrastructure, human resources, training, skill development, equipment and supplies, process of care and patient safety). Information on the same policy document from different data sources (guidelines, reviews, evaluations) was assessed for convergence, in order to triangulate information to minimize bias and increase data validity and reliability. The process was also used for cross-checking of information across different data sources.

## Results

Altogether we analyzed 22 national policy documents, including two policy statements and 20 guidelines on implementation of specific schemes. Seven were comprehensive documents addressing all the MNHFP + A themes. Eleven addressed abortion, 14 each addressed family planning and neonatal health, while 16 dealt with maternal health. The findings below summarize the institutional and socio-economic contextual factors behind the development of policy on quality improvement in MNHFP + A. [See Additional file 1: Appendix 1, a tabular summary of documents included in the policy review.].

### Context/situational factors

#### Population characteristics and need

The scale of demand for public health services in India can be gauged from the fact that India is the world’s second most populous country, home to more than 1.26 billion people [9]. It is also home to 33% of the world’s poorest people, with 26% below the official poverty line in rural as compared to 14% in urban areas [10, 11]. National figures, however, mask the regional disparities observed in social indicators. In India, the maternal mortality ratio is at 167 deaths for 100,000 live births (2011–13), and the neonatal mortality rate is at 29 deaths for every 1000 births (2012) [12]. Additionally, the country has the largest number of women with an unmet need for contraception [13]. MNHFP + A continues to be a health priority with high rates of maternal, neonatal and infant mortality, comparatively low couple protection rates and high incidence of unsafe abortions.

#### Public health and family welfare service provision

Public health services in India are provided free of cost or with a very nominal fee. Service provision in rural areas is through a hierarchy of public health centers, starting with the Sub-Centre at the village level, linking to primary health centers. At the sub-district or block level there are Community Health Centers and at the district level is the district hospital, located in the district headquarters. All public health and family welfare services including abortion are provided through this network of centers, with specializations varying with the level of facility. Unlike rural areas, however, urban areas do not have a well-defined hierarchy of public health centers. There is a concentration of tertiary level centers including district hospitals and in some districts, a medical college. Other than public health centers, India has a robust private health sector, ranging from single physician clinics to large multispecialty hospitals, with tertiary level facilities generally concentrated in large urban centers.

Institutional deliveries in public health facilities are incentivized through a conditional cash transfer scheme called the Janani Suraksha Yojana (JSY). The success of JSY has led to a rise in institutional deliveries from 47% in 2007–08 to 73% in 2010 as per latest available estimates [14, 15]. Quality of care emerged as a key concern during this increase, especially in the light of the expanding utilization of facilities for maternity services. The family welfare services focus on counseling and options for limiting and spacing methods. Governed by a separate Act, induced abortion (or medical termination of pregnancy) in India is legal, but can be provided only in licensed facilities, and only by trained personnel [16].

Multiple political factors influenced MNHFP + A policy formulation in India in the 1990s [Table 2]. Economic reforms initiated in 1991 led to a booming private sector growth in healthcare with little regulation, thereby increasing concerns of quality control, accreditation and safety protocols [17, 18].

**Table 2:** Highlights of policy influences on MNHFP + A quality of care in India

1991	Structural Adjustment Programme launched in the economy – curtailed public social expenditure; leads to rise in private sector health investment in India Quality concerns voiced on growing unregulated private health sector
1992	Launch of Child Survival and Safe Motherhood Programme; setting up First Referral Units for emergency obstetric care
1992–93	First National Family Health Survey held in India – collects in-depth data on maternal and child health
1994–96	The UN Conferences on Population and Development (Cairo, 1994) and Women and Development (Beijing, 1996) held – rise of reproductive rights movement India adopts target-free and RCH approach in 1995; RCH programme introduces integrated MCH, family planning and reproductive health services Quality concerns voiced increasingly but no action strategies formulated
2000–2005	National Population Policy (2000) outlines RCH strategy & sets specific IMR & MMR reduction goals Quality focus in tenth and eleventh plans with strategies for quality assurance & appraisal, including setting up of National Accreditation Board for Hospitals and Healthcare Providers (NABH).
2005	NRHM/RCH-II launched, leading to expanded funding and decentralized programme implementation Quality focus and action strategies in both programmes along with regular monitoring & feedback mechanisms. Quality initiatives include Indian Public Health Standards for quality assurance in primary care; Quality Assurance Committees at district/State level under RCH-II & assistance to states by NABH for quality certification.

The global women’s reproductive rights movement in the early 1990s also influenced the paradigm shift to a client-centered and quality-oriented target-free reproductive health approach [19, 20]. Moreover, large scale surveys produced in-depth demographic and reproductive health data highlighting India’s poor indicators. Pressure to achieve the global Millennium Development Goals also accelerated efforts to improve MCH indicators [20].

The new millennium in India’s health sector therefore saw rising articulation of concerns with the quality of care on the one hand, and the need to revitalize the health sector for accelerated expansion on the other.

### Groups/actors influencing policy formulation

Over the years diverse actors have contributed to the policy making process, from the judiciary to Government, senior experts, academicians, activists and development partners. Under the NRHM, a number of multi-stakeholder task groups on different themes helped detail out its various strategies [21]. The diversity in stakeholders has ensured significant changes from the traditional top-down model of health service provision towards a more decentralized model with greater flexibility to the states. For the first time elements like community based monitoring of services, participatory patient welfare committees and untied funds to facilities for discretionary spending as per their need were introduced.

### Policy content on quality of care

#### Strategies under the NRHM

The National Rural Health Mission (NRHM) (2005–12), the flagship health program of the Government, was launched in 2005 “to carry out necessary architectural correction in the basic health care delivery system” [22]. It significantly increased public expenditure on health, decentralized planning & implementation with greater flexibility to states, community participation and monitoring for accountability.

Under NRHM, the Indian Public Health Standards (IPHS) were constituted as the basis for ensuring that all levels of primary healthcare services across all states adhere to a set of uniform prescribed norms and standards of physical infrastructure, human resources, assured services provided, essential drugs and equipment, treatment procedures and behavior with patients [23]. They also include accountability mechanisms like participatory patient welfare committees, cleanliness, hygiene, blood storage, waste management, patient rights and quality monitoring. Person-centered care indicators included client-friendly reception desk, waiting areas, display and amenities; provision for complaint box to record user complaints and system for addressing them, and the need to ensure patient participation in Rogi Kalyan Samiti (RKS), which is a hospital level patient welfare and management committee [23].

Responding to the need for structured quality improvement in facilities, the Eleventh Five Year Plan (2007–12) provided for setting up of National Accreditation Board for Hospitals and Healthcare Providers (NABH) for accreditation of private and public health centers [24]. Adopting and achieving IPHS norms is less resource intensive than the NABH standards [23].

NRHM acknowledged the denial of healthcare to the community in many ways ranging from deficient facilities (lack of staff, drugs, equipment) to corruption, refusal of treatment on account of inability to pay fees, insulting or discriminatory behavior of staff and inadequate attention given to the patient resulting in poor quality of care. Community action mechanisms were designed to address these issues. Participatory forums like RKS and Village Health Sanitation and Nutrition Committees (VHSNCs) were established for participatory community action on health and its determinants. The framework for district action planning under NRHM has quality of care as one of its key components, comprising technical competence, interpersonal communication, client satisfaction, accountability and redress mechanisms.

#### Strategies under RCH-II

The second phase of the Reproductive and Child Health Programme (RCH-II) (2005–10) was also launched in 2005, focusing on reproductive and child health as a health priority. Following a Supreme Court of India directive in March 2005, a Working Group on Quality Assurance in RCH-II was set up to design an internal and independent quality monitoring for RCH services. A system of quality assurance committees to monitor quality of RCH services at the district level, coordinated by a State level such committee was envisaged.

The RCH-II QA strategy included monitoring of service quality at the health center level and systematic efforts to improve quality through training, behavior-change communication, evaluation and feedback. Elements of quality assessed included access to services, facility infrastructure, transport arrangements, communications, equipment and supplies, professional standards, technical competence and continuity of care [25]. All the states in the country have now constituted State and District level Quality Assurance Committees to monitor quality of care in RCH services.

National and state level technical assistance agencies were established for building capacity (managerial, technical, human resources, knowledge management, information sharing & convergence) on healthcare management and qualitative strengthening of institutions. Infrastructure of training institutions, especially for obstetrics-gynecology and pediatrics, were also to be improved. Protocols for quality assurance including clinical guidelines for RCH care/counselling were to be put in place by the Government and treated as minimum standards of quality. Monitoring methods could include surprise checks, client satisfaction surveys, mystery-client surveys, sample FGDs and checklists for compilation of service quality data. These were expected to assess person-centered care in public facilities.

For the private providers the Government postulated laying quality standards and encouraging participation of professional medical associations to contribute effectively to developing quality control standards. State governments will provide interim accreditation, monitor & regulate private sector & build capacities of district units.

#### Quality assurance in urban facilities

In 2012, the NRHM was transformed to the National Health Mission with an urban component called National Urban Health Mission (NUHM) as well. NUHM envisaged implementation of IPHS and quality assurance cells in all urban public facilities [26]. It also defined parameters for engaging NGOs for quality monitoring. The private sector has a significant presence in urban areas and the NUHM talks about identifying private partners and tapping their skills to improve the quality and standard of health among the urban poor, by capitalizing on the skills of potential partners, encouraging pooling of resources, and supplementing public resources. Compliance with quality standards is a critical criterion for selection of private facilities to partner with the public sector. It also envisaged capacity building of public and private providers for providing quality services, ensuring citizen charters in facilities and encouraging development of standard treatment protocols [26].

#### Maternal death and near-miss reviews

Apart from quality standards, the system of maternal death review was institutionalized through guidelines in 2010 in order to identify factors that led to a maternal death. This helped evaluate the entire process of care received by the woman, and through this analysis to see what can be improved to avoid such deaths in the future. Similarly, guidelines were also issued for maternal near-miss audit in 2014, when it was realized that many cases of complications or near misses could also suggest measures to improve quality of care in order to minimize such events.

#### RMNCH + A strategy

The Government of India launched the Reproductive, Maternal, Newborn, Child Health and Adolescent (RMNCH + A) strategy in 2013 for integrated provision of care on these aspects. Consequently the Operational Guidelines on Quality Assurance in Public Health Facilities were brought out in 2013. Program reviews revealed that states focused mostly on creating IPHS specified infrastructure and deploying recommended human resources and the user’s perspective was often overlooked [27]. The need to create a sustainable quality improvement system for public health facilities which not only delivers good quality services but is also so perceived by the clients was realized [27]. The guidelines have been prepared with this perspective defining relevant quality standards, a robust system of measuring these standards and institutional framework for its implementation [27].

The Operational Guidelines on Quality Assurance in Public Health Facilities were brought out in 2013. The guidelines define quality in terms of both technical quality (clinical protocols, infection control, and emergency response) and service quality (prompt service delivery, courteous behavior of staff, hygiene and cleanliness, privacy and dignity). They describe the role and jurisdiction of quality assessment teams at the national, state and district level. They also outline specific ‘areas of concern’ on which the quality assessment focusses, including: service provision, patient rights, inputs, support services, clinical services, infection control, quality management and outcome indicators. The guidelines are accompanied with assessor’s handbooks for quality assurance in district hospitals, describing minimum standards for infrastructure, equipment, counselling and other services in all departments [27].

One of the key areas is strengthening competency based training of healthcare providers for RMNCH + A services. “Skills Labs” were envisaged at the district and sub-district levels to facilitate acquisition/reinforcement of key standardized technical skills and knowledge by service providers for RMNCH + A services. The specific skill competencies include antenatal care, intra-natal care, complication management, new born care, family planning, infection prevention, counselling and documentation. The guidelines make a conscious effort to move away from the IPHS focus on infrastructure to more process oriented measures, including development of standards, skill building and robust quality monitoring. The strategy realized that ensuring quality of services and more importantly user’s perspective was often overlooked in the current quality assurance system [28].

#### Comprehensive abortion care

The liberalization of abortion laws in India began in the 1960s in the context of extremely high maternal mortality resulting from unsafe abortions. A Government Committee examined the socio-cultural, legal and medical aspects of the issue, and in 1966 recommended the legalization of abortion in the country, to ensure women’s health on both compassionate and medical grounds. Finally, in 1971, the Medical Termination of Pregnancy Act was enacted by the Parliament of India, legalizing abortions till 12 weeks of pregnancy [29]. In case of pregnancies exceeding 12 weeks and but less than 20 weeks, termination requires the opinion to two doctors [30]. However, despite having one of the broadest abortion laws globally, unsafe abortions outnumber legal procedures and contribute to maternal deaths in India [30]. The quality of abortion care, therefore, is also a key area of concern that policies aim to address. Under the government’s current RMNCH + A strategy, every delivery point or facility providing delivery services should be able to provide comprehensive abortion care.

Under the RMNCH + A strategy, guidelines for Comprehensive Abortion care were issued in 2014, under the purview of the Medical Termination of Pregnancy (MTP) Act (1971) [16]. The objective is to serve as a guide for program managers and service providers for providing woman centric comprehensive abortion care at public health facilities. The guidelines prescribe attention to certain aspects of care while providing abortion services - privacy and confidentiality, polite, courteous and non-judgmental staff, ensure that reproductive rights are respected when providing services, clean and hygienic surroundings, availability of uninterrupted power and water supply, clean toilets and assured referral linkages. Checklists and lists of essential equipment are also provided. The guidelines are also accompanied with (a) Trainer’s and Provider’s Manuals (b) Power point presentations (c) Posters on technical content (d) Manual Vacuum Aspiration training material and (e) Operational guidelines for program managers to monitor and supervise the services with the goal to strengthen provider skills in performing safe abortions, pre and post abortion counseling and post training supportive supervision and follow up. Guidance includes women centric comprehensive abortion care covering clinical procedure, counselling, post-abortion contraception and equipment requirements.

## Discussion

Findings show that in recent years India has developed a comprehensive set of guidelines and strategies that are driving quality assurance and improvement in MNHFP + A services in the country. NRHM and RCH-II for the first time laid out a long-term vision of improving quality, access, coverage, and assuring service availability and effectiveness [20]. Quality guidelines and strategies exist relating to infrastructure and commodities, human resources, competencies, translation of skills into practice, and accountability and commitment. Strong regulatory systems have also been designed to monitor quality of service delivery both in the government and the private sector. However, person-centered care is not explicitly detailed (except to some extent in comprehensive abortion care), and is currently limited largely to directives on maintaining patient privacy, or taking client feedback and setting up a system of grievance redress [31]. Aspects such as promptness of care, information sharing and respectful behavior need to be incorporated more strongly in the policies.

Evidence from program evaluations highlights gaps in quality of care affecting utilization of facilities. The conditional cash transfer strategy to increase institutional deliveries has not conclusively impacted outcomes like maternal mortality [32]. This unexpected shortcoming has been associated with persisting quality of care challenges including chaotic delivery environment, not conducive to safe, women-friendly care, lack of routine skilled care provision, frequent abuse or neglect of women during delivery and demand for bribes [32, 33]. The net money transfer was also not appreciable [33, 34]. Evidence also points to the need for considering emotional and psychological cost to women of delivering in hospitals while incentivizing birth outcomes [34].

Person-centered care is one of the key reasons why private facilities are increasingly gaining precedence over public ones. A study in Mumbai’s informal settlements found that poor perceptions of public facilities combined with concern for a positive experience of care and health outcomes often caused residents to utilize tertiary hospitals or private sector facilities [35]. Users valued good behavior along with staff availability, comprehensive services, good physical infrastructure while opting for private facilities, and cited politeness, understanding and cooperation towards patients and relatives as the major qualities of good health providers (35). The Government’s own reviews and evaluations point out the need to focus more attention on making the system more outcome-oriented and responsive to patient’s needs, like courteous behavior by staff and explanation of diagnosis, treatment and drugs to patients—these do not appear to be addressed, and have emerged as one of the major reasons for non-utilization of public facilities [36].

Person-centered care is hindered by the persistence of structural challenges in health facilities. Poor infrastructure and supplies, lack of adherence to protocols, staff absenteeism, lack of prompt treatment, general apathy, disrespectful behavior, difficulty in availing incentives and demand for bribes are some of the diverse quality challenges faced by users in public facilities [32, 33, 37]. Evidence from public hospitals in urban areas points at the need to address the process of care after infrastructure and supply needs have been addressed. User and provider perspectives both identify infrastructure, human resources, supplies and medicine as priority areas of quality improvement in the facility [33]. This is the basic requirement for a facility to be fully functional. Once that is met, the patient prioritizes other aspects of quality like waiting time and information sharing.

In the light of our findings, we recommend that person-centered care should be addressed more comprehensively in the current policy/strategies. Evidence shows that women value aspects like emotional support, information sharing, privacy and prompt care as much as availability of health providers, appropriate clinical care, cleanliness and cost [31]. More detailing of norms for respectful behavior, information sharing, promptness, emotional support and other norms need to be incorporated in the policy, with a clear plan of public and staff awareness and orientation, implementation, monitoring and feedback. Policy also could incorporate mechanisms to embed patient-centered care in the provision of services, such as constituting quality circles and a quality counsellor for the staff at hospitals to help orient on patient-centered care, establishing standards for prompt attendance of patients and publicly displaying behavioral norms in the charter of patient rights. Patient welfare committees at hospitals could also collect client feedback on patient-centered care. A system of grading and recognizing facilities that provide good person-centered care could be introduced to encourage adoption of such practices. Community education could be incorporated into the policy to make users more aware of the care entitled to them and encourage them to demand the same from the system.

The Government of India has expanded its scope of quality monitoring to cover MCH services at facilities, including assessing the quality of training provided to staff, and has increased the overall financial commitment for it [38]. The emphasis on training and skill building in the guidelines is much needed to ensure appropriate clinical care adhering to protocols and quality norms, which is a critical aspect of person-centered care.

Training should also incorporate aspects of the person-centered care like interpersonal behavior, information sharing, ensuring privacy and appropriate counselling. Also, simple tools to audit the quality of care at the facility level should be provided, with components on person-centered care as well. These tools should be easy to use, preferably to be administered by internal quality improvement committees on a periodic basis. Emerging issues also need to be addressed through constructive feedback and mentoring, rather than any punitive measures.

Lastly, evidence also shows that existing policy provisions for person-centered care in the policy/strategies are not being adhered to effectively. Implementation hurdles to strategies on person-centered care are a persistent challenge. Patient satisfaction, for example, is indispensable to quality improvement with regard to design and management of healthcare systems [39]. However, currently quality improvement initiatives do not regularly assess patient satisfaction with services or disrespectful and abusive behavior of service providers [40]. Community feedback and monitoring mechanisms, which have the potential for addressing such issues, are also not functioning effectively in all regions.

In public hospitals where NRHM has successfully addressed infrastructural issues, the time is appropriate to focus on patient-centered care. But this has not been fully achieved in all facilities. Heavy caseloads in tertiary public hospitals have undermined the structural improvements undertaken there. Shortage in human resources has also increased over the years [27]. Thus, while policy needs to be strengthened on some aspects of patient-centered care, at the same time existing gaps in structural dimensions of quality of care need to be addressed to provide an enabling environment for achieving patient-centered care.

Our study is a preliminary effort in examining patient-centered care in MNHFP + A policies in India. Our findings pertain to the Indian context and may not be generalizable to other regions. Another limitation of our study is that it is based entirely on desk review, not supported by key informant interviews or policy evaluations that could have possibly strengthened it.

## Conclusion

The post-NRHM era in India has been marked by expanding strategies for quality assurance and improvement in the critical and priority areas of maternal and newborn health, family planning and abortion. We conducted a retrospective policy review and found that while increasing the coverage of services has led to increased utilization, person-centered care is a relatively neglected area in terms of both policy and practice. The component of patient-centered care in policies needs to be strengthened, possibly with stronger implementation and monitoring mechanisms to ensure compliance [40]. We recommend strengthening of policy on person-centered care, with the caveat that unless implementation hurdles in existing policy are addressed, such as infrastructure, human resources and supplies, policies on patient-centered care may not be effectively implemented. These factors are essential to create an environment conducive to the practice of patient-centered care by providers. But at the same time priority to person-centered care cannot be compromised as it is fundamental to upholding patient rights and dignity. Both infrastructure and treatment norms that affect patient experience can and should be addressed in parallel.

Addressing some of the issues highlighted in our analysis is pivotal to improving utilization of care, adherence to treatment, continuity and follow up and also ultimately impacting positively the MNHFP + A outcomes in the country.

## Additional file

Additional file 1: Appendix 1: Summary of policy documents included in the review. The additional file contains a tabular summary of policy documents included in the policy review undertaken for this study. Details listed include name, year and publisher of the document, geographic or population coverage, aim and objectives of the document and key points in the implementation plan that relate to quality improvement. (PDF 238 kb)

## Abbreviations

IMR: Infant Mortality Rate
IOM: Institute of Medicine;
IPHS: Indian Public Health Standards
MCH: Maternal and Child Health
MMR: Maternal Mortality Rate
MNHFP + A: Maternal, Neonatal Health, Family Planning and Abortion
MTP: Medical Termination of Pregnancy
NABH: National Accreditation Board for Hospitals and Healthcare Providers
NRHM: National Rural Health Mission
NUHM: National Urban Health Mission
QAC: Quality Assurance Cells
QC: Quality Circle
RCH: Reproductive and Child Health
RKS: Rogi Kalyan Samiti
RMNCH + A: Reproductive, Maternal, Newborn, Child Health and Adolescent
SDG: Sustainable Development Goal
VHSNC: Village Health, Sanitation and Nutrition Committee
