# Supplementary material for: Putting women at the center: a review of Indian policy to address person-centered care in maternal and newborn health, family planning and abortion
Source: BMC Public Health. 2017 Jul 14;18:20. doi: 10.1186/s12889-017-4575-2 (PMC5513115; doi:10.1186/s12889-017-4575-2)
Supplement: Additional file 1: — Appendix 1: Summary of policy documents included in the review. The additional file contains a tabular summary of policy documents included in the policy review undertaken for this study. Details listed include name, year and publisher of the document, geographic or population coverage, aim and objectives of the document and key points in the implementation plan that relate to quality improvement. (PDF 238 kb) [file 12889_2017_4575_MOESM1_ESM.pdf]

Appendix 1: Summary of policy documents included in the review

Name; Department; Year	Coverage (Geographic; Population Groups)	Aim and Objectives	Key points in implementation plan relating to Quality Improvement (QI)
The Planning Commission, 12th Five Year Plan, 2012	Pan- India, includes district level health care providers	Reducing MMR to 100; reduction of Anaemia among women aged 15-49 years to 28 percent; reduction of Total Fertility Rate to 2.1.	1. Revision of Indian Public Health Standards. 2. Setting up of in-house quality management systems at government and publicly-funded facilities. 3. Expanding access among under-served populations by increasing the existing Mobile medical units. 4. Quality standards for facilities should be taken as guiding principle for sanctioning posts, which would indicate the maximum staff that can be posted. 5. Careful monitoring of the Quality of Care being provided in routine institutional deliveries. 6. Creation of accessible grievance redressal mechanisms. 7. Completion of training of ASHAs and retraining of the existing cadre of workers as Male Multi-Purpose Workers, AWW and

			ANMs, to make them relevant to local needs, and for their own upward mobility.
Skills Lab Operational Guidelines: Strenghtening Competency based training of healthcare providers for RMNCH + A services, NRHM/MHFW, 2013	District level, healthcare providers	Building the capacity of healthcare workers through specialized competency-based skills training, in order to reduce the MMR	1. Setting up of "Skills Lab". 2. Skills Lab will facilitate acquisition / reinforcement of key standardized technical skills and knowledge by service providers for RMNCH+A services (antenatal care, intra-natal care, complication management (MNH), new born care, family planning, infection prevention, counselling and documentation). 3. Ensure the availability of skilled personnel at health facilities. 4. Improve quality of pre-service training. 5. Provide continuing nursing and medical education.
A Strategic Approach to RMNCH + A in India, NRHM/MHFW, 2013	Pan- India, includes district level health care providers	Increase facilities for perinatal care by 100%; Increase proportion of births conducted by skilled attendants at an	1. Emphasis on quality, focussed on the addition of infrastructure and human resources, equipment, drugs and supplies. 2. Ensure public services an affordable range. 3. Women, mothers, newborn, child and adolescent friendly services,

		annual rate of 2% from the baseline of 76%; Increase proportion of mothers receiving post-natal care at an annual rate of 7.5% from the baseline of 45%; Reduce unmet need for family planning methods among eligible couples, married and unmarried, at annual rate of 8.8% from the baseline of 21%.	with no social barriers keeping out the poor and marginalized. 4. Standardized quality assurance system across all the States. Clearly defined roles and responsibilities for each level in QA system. These will include (1) Central Quality Supervisory Committee; (2a) State Quality Assurance Committees, (2b) Quality Assurance Cell and (2c) Full time quality assessors; (3) District Quality Assurance Committees; and (4) Quality Circles at the District Hospital level.
Guidelines for Sub District/ Sub Divisional Hospitals, Indian Public Health Standards, 2012	District level healthcare providers	1. Provide comprehensive secondary health care (specialist and referral service) to the community	Standardization of quality norms across district hospitals, improvement of infrastructure and enhanced training for healthcare workers. Quality control mechanisms: 1. Internal Monitoring-medical, technical, financial and

		through the sub district hospital. 2. Achieve and maintain acceptable standard quality of care. 3. Make the services more responsive and sensitive to the needs of the people.	disaster preparedness audit, monitoring of accessibility and equity issues. 2. Death review- Fortnightly review of all mortality that occurs in the hospital, including maternal deaths. 3. External Monitoring- Monitoring by RKS, local bodies, service/performance evaluation by independent agencies, District Monitoring Committees under NRHM, public hearings. 4. Citizens Charter- Each Sub-district hospital should display a citizen's charter indicating the services available, user fees charged, if any, and a grievance redress system.
IUCD Reference Manual for Medical Officers and Nursing Personnel, 2013	Programme managers, trainers and service providers	Latest information on IUCD to provide high quality services that are safe and client centred. Institutional deliveries as opportunities to	1. Service Delivery Guidelines: Counseling on post-partum family planning; counseling on IUCD advantages, limitations, effectiveness and side effects; explanation of procedure for insertion and/or removal of the IUCD; screening as per WHO Medical Eligibility Criteria. 2. Guidelines on

		counsel pregnant women and IUCD services to women in postpartum period. Training on IUCD services with a long-term plan of repositioning the IUCD in India's Family Welfare Programme as a spacing method.	counselling: GATHER Approach- G Greet the client respectfully, A Ask them about their family planning needs, T Tell them about different contraceptive options and methods, H Help them to make decisions about choices of methods, E Explain and demonstrate how to use the methods, R Return/refer; schedule and carry out a return visit and follow up. 3. Quality guidelines - HR requirements, client focused service, management systems, infection prevention, insertion and removal process, follow up visits.
Guidelines for District Hospitals, Indian Public Health Standards, 2012	District level healthcare providers	1. To provide comprehensive secondary health care (specialist and referral services) through the District Hospital. 2. To achieve and maintain an	1. Guidelines on patient safety and infection control, health worker safety, physical infrastructure, human resource requirements and equipment. 2. Quality control guidelines including management information systems, internal audits, medical audits and death reviews (especially for maternal deaths). 3. Provisions for grievance redressal-

		acceptable standard of quality of care. 3. To make the services more responsive and sensitive to people's needs.	prominent display of citizen's charter in local language indicating the services available, user fees charged, if any, and a grievance redressal system.
Assessors Guidebook for Quality Assurance in District Hospitals Volume I, Maternal Health Division, NRHM/MHWF, 2013	District level assessors and health care providers	Standards and measurable elements including checklists for quality assessment of district hospitals.	Describes the minimum standards required in district hospitals, including those for infrastructure, equipment and counselling services in various wings: accident and emergency, outpatient department, labour room, maternity ward, paediatrics ward, sick newborn care unit, nutritional rehabilitation centre, operation theatre, post-partum unit.
Assessors Guidebook for Quality Assurance in District Hospitals Volume II, Maternal Health Division,	District level assessors and health care providers	Standards and measurable elements including checklists, for quality assessment of district hospitals.	Describes the minimum standards required in district hospitals, including those for infrastructure, equipment and counselling services in various wings: ICU, Indoor Patient Department, Blood Bank, Laboratory services, Radiology and USG, Pharmacy, Auxiliary services,

NRHM/MHWF, 2013			Mortuary, and general administration.
Operational Guidelines on Quality Assurance in Public Health Facilities, NRHM/MHFW, 2013	National, State and District level	'Road-map' for implementing quality assurance at the state level. The 'quality assurance' approach to help improve the quality standards of public health facilities in all states. Includes technical quality (clinical protocols, infection control, emergency response) and service quality (prompt service delivery, courteous behaviour of staff, hygiene and	Describes the role and jurisdiction of quality assessment teams at the national, state and district level. It also outlines specific 'areas of concern' which the quality assessment focusses including: service provision, patient rights, inputs, support services, clinical services, infection control, quality management, outcome indicator.

		cleanliness, privacy and dignity).	
Postpartum IUCD Reference Manual, NRHM/MHFW, 2010	Healthcare workers	Reducing the risks associated with the post partum period; Postpartum period is one of the critical times when both woman and newborn need a special and integrated package of health services as morbidity and mortality rates are quite high during this period and also the women are vulnerable to unintended pregnancy. Studies show that	Provides essential information to healthcare workers about post partum family planning, post partum IUCD, family planning counselling, infection prevention, management of potential problems and follow- up care. Includes checklists for IUCD insertion and removal, skills list for employees, and performance standards for post partum IUCD clinical services, among others.

		pregnancies taking place within 24 months of a previous birth have a higher risk of adverse outcomes like abortions, premature labor, postpartum hemorrhage, low birth weight babies, fetal loss and maternal death.	
Maternal Near Miss Review: Operational Guidelines, Maternal Health Division/MHFW, 2014	Healthcare workers	Investigating cases of life threatening obstetric morbidity or Maternal Near Miss (MNM) would help to bring further improvements to the maternal	Includes details on training and sensitization of employees on how to review MNM. Steps include: 1. Identifying if a case satisfies inclusion criteria, 2. Identify adverse effects, 3. For each adverse effect elaborate possible conditions or disorders, 4. the result of the investigation which make women fall under MNM category are identified, 5. The

		helath programme. 1. To identify the technical and non-technical causes of MNM. 2. To identify the health system response to maternal emergencies. 3. To identify the gaps and contextualise corrective measures to be taken in the health care system. 4. To provide regular feedback and response needed to achieve the goals. 5. Identify best practices	intervention that saved the mother is recorded. Detailed case forms are provided in the report.
Operational Guidelines for Facility based	Healthcare workers	F-IMNCI is the integration of the Facility based Care	Includes detailed guidelines on training of healthcare personnel when providing neonatal care:

Integrated Management of Neonatal and Childhood Illness (F-IMNCI), NRHM/MHFW, No date		package with the IMNCI package, to empower the health personnel with the skills to manage new born and childhood illness at the community level as well as the facility level. It helps to build capacities to handle referrals taking place from the community. The implementation of F-IMCI strategy will help improve the performance and quality of health workers. The critical element of this	modules for physicians, physician chart book, photo book for physicians, facilitator guide, indoor - out-door patient guide. Around 50% of the training time is allocated to case management and counselling sessions. The guidelines also include in-depth checklists covering essential equipment.

		strategy is the evidence-based integrated approach with a focus on new born and child hood illness. This package will also help address the acute shortage of pediatricians at facilities.	
Facility based Newborn Care operational Guidelines, NRHM/MHFW, 2011	National, State and District level	Operational guide has been developed to facilitate the planning, establishment, operationalization, and monitoring of newborn care facilities at various levels of the public health care system.	The first section of the guide focuses on specifications and processes related to establishment of new facilities, while the second provides technical guidance on how to manage sickness among newborns with detailed checklists and explanation of best practices.

Facility based Newborn Care Neonatal Resuscitation Module, NRHM/MHFW, 2014	Healthcare workers	Training manual to enable health workers to successfully resuscitate newborns.	Provides vital information including: the physiological changes that occur at birth, the sequence of steps to be followed at resuscitation, risk factors which can predict which babies may need resuscitation, equipment and personnel requirements for resuscitation.
Basic Newborn Care and Resuscitation Program Manual, NRHM/MHFW, No date	Healthcare workers	The Basic New born Care and Resuscitation program developed by the Ministry of Health and Family Welfare will help develop skills of health providers to address birth asphyxia and other causes of mortality at birth. The two day training envisaged under this	Improving skills of workers by providing them with essential skills on newborn resuscitation. Also provides information about significant equipment.

		program, would be able to enhance the skills of health providers and especially the birth attendants at facilities. It is estimated that this skill based training when put in place in the States can prevent approximately 1- 2 lakh newborn deaths every year.	
Facility Based Newborn Care Training Module for Doctors and Nurses, NRHM/MHFW, 2014	Doctors and Nurses	Training module for Doctors and Nurses in providing essential care to newborns.	Includes detailed description of clinical procedures to tackle potential risks to newborns, and demonstrates the correct use of equipment. Also provides a guide on communication skills essential in neonatal care.
Guidelines for Janani-Shishu	Healthcare workers and	Guidelines for the States to ensure	Detailed description of essential actions to ensure smooth

Suraksha Karyakram, NRHM/MHFW, 2011	administrative staff at the State and District level	effective implementation of JSSK scheme.	implementation of JSSK, including elements related to grievance redressal and monitoring & follow up.
Comprehensive Abortion Care Training and Service Delivery Guidelines, NRHM/MHFW, 2010	healthcare workers	Guidelines on providing comprehensive abortion care	Details the process of providing counselling around the procedure; pre-procedure, post procedure, if the woman has been referred to a higher level facility, counselling during a follow up visit. Also provides troubleshooting suggestions and information about post abortion contraceptive suggestions.
Guidelines for Antenatal Care and Skilled Attendance at Birth by ANMs/LHVs/SNs, NRHM/MHFW, 2010	State and District level healthcare workers and administrators	Guidelines to update the skills and knowledge of state and district program officers, trainers, ANMs, LHVs, SNs in various technical interventions.	Provides technical information related to both clinical skills and use of equipment. Emphasizes the importance of quality of care and counselling services; "Respectful communication and genuine empathy are the most important elements of quality maternal care."

Guidelines for operationalising a Primary Health Centre for Providing 24-Hour Delivery and Newborn Care Under RCH-II, Maternal Health Division/MHFW, 2005	State and District level administration	These guidelines are meant to assist the states in formulating their own implementation plans regarding the operationalization of 50% of the Primary Health Centers and all the Community Health Centers as 24 hour delivery and newborn care service locations.	Provides details of essential HR, equipment and technical expertise required to setup PHCs and CHCs.
Operational Guidelines Comprehensive Abortion Care Services, NRHM/MHFW, 2014	Pan India, health care workers and administrative staff	Guide for program managers and service providers for providing woman centric comprehensive abortion care at health facilities	Describes the following components of care which must be present: privacy and confidentiality, polite, courteous and non-judgemental staff, ensure that reproductive rights are respected when providing services, clean and hygienic surroundings, availability of 24x7

		within the public sector in India.	water supply, uninterrupted power supply, and clean toilets, assured referral linkages.
Providers Manual Comprehensive Abortion Care Services, NRHM/MHFW, 2014	Healthcare workers	Purpose of this manual is to: Provide standardized training material including teaching aids to all states for CAC services; Strengthen skills of Medical Officers for performing safe MTPs, the skills of ANMs and Nurses in pre and post abortion counselling and post training supportive supervision and follow up; Assist in strengthening the	Includes training material on women centric comprehensive abortion care covering clinical procedure, counselling and equipment.

		currently available abortion care services and improving the overall quality of care and Promote the concept of woman-centric care in the provision of abortion services.	
